# Tumor seeding following CT- guided transthoracic needle biopsy in lung cancer. A case report

**DOI:** 10.1186/s12890-023-02712-0

**Published:** 2023-10-24

**Authors:** Thomas Melzer, Caroline Maria Hackl, Julia Walter, Jürgen Behr, Amanda Tufman, Pontus Mertsch, Diego Erich Kauffmann-Guerrero, Kathrin Kahnert

**Affiliations:** 1 Department of Medicine V, University Hospital Munich (LMU), Comprehensive Pneumology Center Munich (CPC-M), Member of the German Center for Lung Research (DZL), Ziemssenstr. 1, 80336 Munich, Germany; 2 MediCenter Germering, Germering, Germany

**Keywords:** Lung cancer, Transthoracic needle biopsy, CT-guided lung biposy, Tumor seeding, Case report

## Abstract

As a result of advances in the treatment of lung cancer, the life expectancy of lung cancer patients has improved significantly, but it remains the leading cause of cancer death worldwide. For decades, most of the initial tumor biopsies have been obtained by bronchoscopy or computed tomography (CT)-guided transthoracic lung biopsy without concerning reports of cancer seeding following the latter. In this case report we discuss the patient history of a 56-year old women with low-differentiated squamous cell lung cancer who developed tumor seeding following a CT-guided transthoracic biopsy 11 months after the intervention. This is put into context reviewing former and current literature.

## Plain language summary

In this case report, we summarize the finding of a transthoracic tumor seeding following a CT-guided lung cancer biopsy in a lung cancer patient. Due to advanced tumor stage independent from the described tumor seeding, this did not change the individual prognosis. However, there are new studies indicating that this side effect is increasingly found in lung cancer patients especially diagnosed at early tumor stages. With this case report, we would like to draw the attention of practitioners to this issue and highlight the need for future studies to assess the long-term effects of CT-guided biopsies versus bronchoscopic biopsies on local tumor progression as well as overall survival.

## Background

Lung cancer is the most common cause of cancer related death worldwide [1], which is a consequence of a decades-lasting increase of incidence mostly amplified by lifestyle e.g. smoking, pollution and global aging [2]. Tackling this health challenge, there has been recent advance in lung cancer treatment leading to increased patients’ prognosis [3]. While 5-year relative survival hardly exceeded 10% in 1975, it is estimated by 35% in 2020 and further refinement is foreseeable [4]. Early detection of lung cancer using lung cancer screening is the next major milestone in improving prognosis [5]. It is already established in countries such as the USA [6] and several provinces of Canada [7], additionally its introduction is being prepared in other countries e.g. Germany [8]. With the establishment of CT screenings in industrialized countries, more pulmonary nodules will be diagnosed and require assessment [9]. Considering increasing life expectancy of lung cancer patients due to improved therapy and earlier diagnosis, the question arises, whether late complications may result from diagnostic procedures that were previously unrecognized, for example seeding metastasis after CT-guided transthoracic biopsy.

Tissue biopsy for histological confirmation is currently mostly obtained by bronchoscopy or CT-guided transthoracic needle biopsy based on size and location of the pulmonary nodule [10]. Previous investigations did not show frequent tumor seeding into chest wall following CT-guided transthoracic biopsy [11] [12]. The largest study, conducted in the 1990s, reported only 8 cases of tumor seeding in the follow-up of 4365 patients including all cancer stages. The follow-up interval covered 5 to 93 months after the procedure. [13] Nevertheless, two recently published studies suggested an increase in the prevalence of tumor seeding following CT-guided biopsy. Gwan Kim et al. reported pleural recurrence in 40 of 415 cases (9,6%) [14], Hong et al. in 118 of 1158 cases (10,2%) [15] with stage I lung cancer.

Interestingly, chest wall implantation in pleural mesothelioma in biopsy tracts is known widely among pneumologists [16]. Even prophylactic radiotherapy was established for many years [17] but currently questioned in the absence of evidence for a beneficial effect in terms of tumor seeding [18].

However, metastasis by iatrogenic procedures can have serious clinical impact and consequences even in advanced tumor stages also affecting patients’ prognosis. In the following, we illustrate this by case of a patient attending our clinic with suspected post-interventional tumor seeding in lung cancer.

## Case presentation

**Fig. 1:**
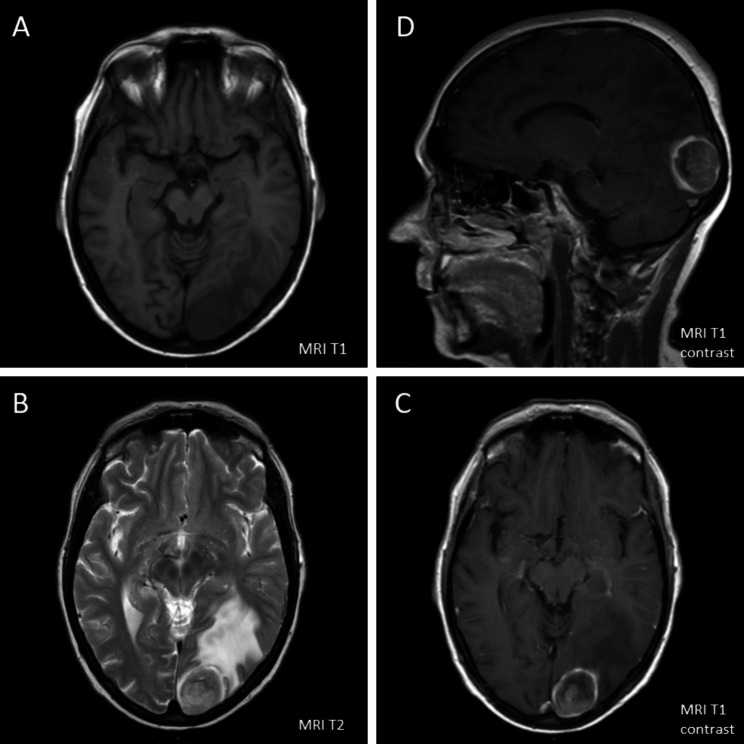
Brain MRI-Imaging initially conducted because of right field hemianopsy. On the left side T1 and T2 sequence without contrast agent (**A, B**), on the right side T1 sequence after contrast showing an occipital lesion with circular enhancement (**C**, reconstructed data **D**). Besides, a beginning brain edema of left occipital lobe could be found

**Fig. 2:**
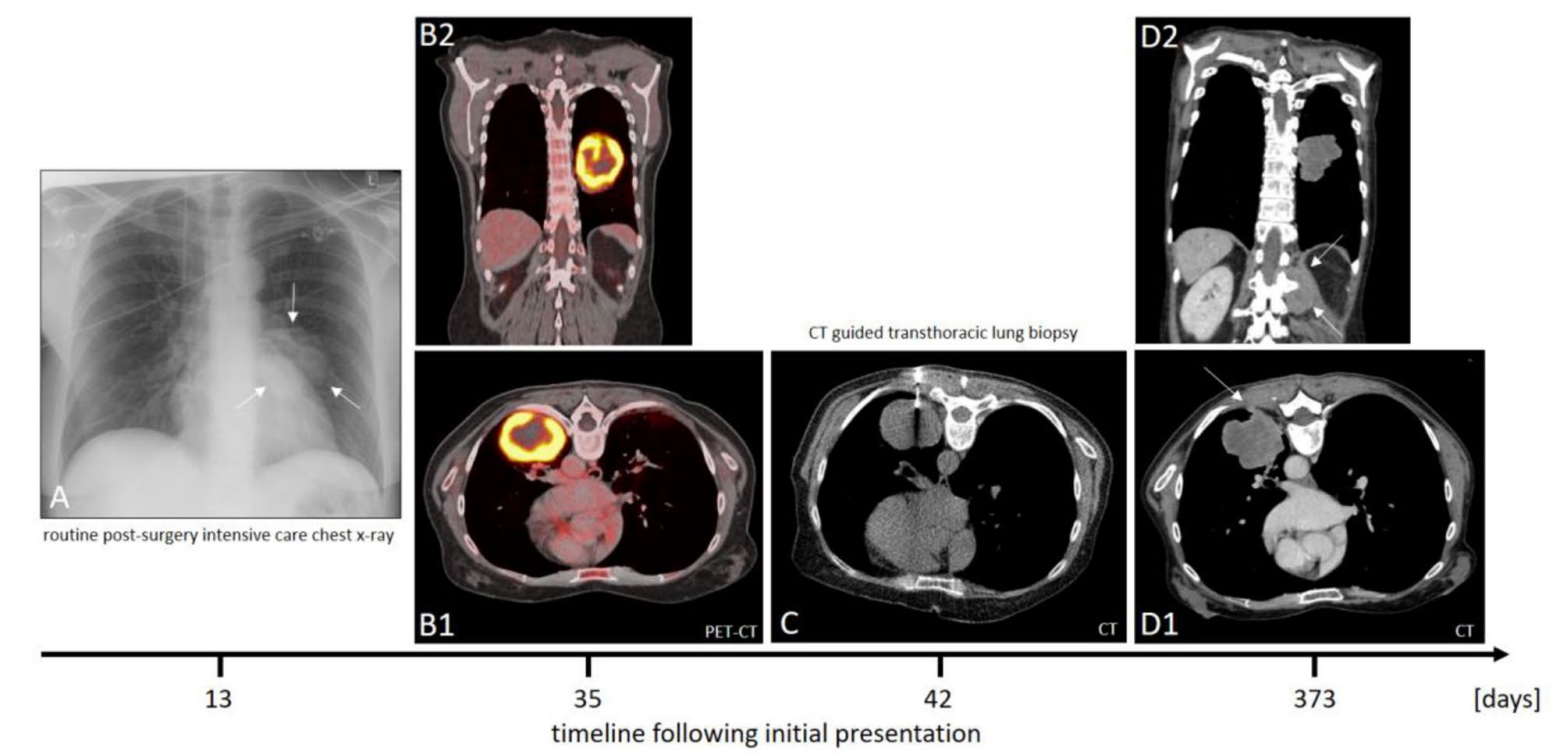
Brain surgery with diagnostic tumor resection had been executed 13 days after first presentation. A post surgery X-ray for routine purpose showed a suspicious mass in left pulmonal midfield (**A**). Diagnostic workup was followed by CT scan (not shown) and PET-CT. Showing strong glucose uptake (**B1-2**), a transthoracic biopsy was performed (**C**). Approximately one year after first imaging, a last CT scan lead to the expectation of tumor growth lead by biopsy channel (**D1**) and forming a beginning soft tissue metastasis. However, a new second muscle and connective tissue invasion was found below infiltrating spinal channel (**D2**)

The 56-year old ex-smoking female was initially diagnosed with low-differentiated squamous cell lung cancer and brain metastasis (Fig. 1) in December 2015. After neurosurgical resection of the symptomatic brain metastasis, the staging was completed with chest X-ray (Fig. 2A), computed tomography (CT) and positron emission tomography (Fig. 2B1 and B2) resulting in stage cT3, N3, M1c [19]. Tissue samples from brain metastases were not suitable for adequate characterization of lung carcinoma due to regressive changes, necessitating repeat histology acquisition. Therefore, a CT-guided tumor biopsy (Fig. 2C) was successfully conducted six weeks after resection of the brain metastasis. In the histopathological workup of the tumor biopsies, initially small cell lung cancer was suspected and due to high tumor burden an immediate treatment with Cisplatin/Etoposide was started. The final histopathological work-up revealed the diagnosis of a low-differentiated squamous-cell lung cancer and the chemotherapeutic regimen was switched to a combination treatment with Carboplatin/Gemcitabine. The systemic tumor treatment was combined with a post-surgery whole brain radiation and following stereotactic radiation of new brain metastasis. 12 months after inital diagnosis, tumor progression at the primary tumor site, pulmonary metastasis, new hepatic lesions as well as progression of brain metastasis led to clinical deterioration (Fig. 2D2). Furthermore, a new tissue branch intersecting tumorous tissue and biopsy channel could be seen, suggesting tumor seeding (Arrow Fig. 2D1).

Additionally, a second completely new soft tissue and muscle metastasis was found next to the left lumbal spine (Fig. D2) and compressing spinal channel at nearly the half of the diameter causing the patient’s back pain next to the findings adjacent to the primary tumor. Suffering from end stage cancer, the patient demanded for discharge. According to bavarian cancer register data, she died three weeks later.

## Discussion and conclusions

In this case discussion we review a 56-year-old female lung cancer patient with adjacent tumor seeding in the area of the previous CT-guided biopsy channel.

Lung cancer remains the leading cause of cancer death worldwide [1], yet a distinction must be made between patients diagnosed at early stages who have an average 5-year-survival above 60% compared to stage IV patients with lower than 10% [20] Furthermore, the mutation status has a significant impact on the overall prognosis [21]. Due to a significantly improved overall prognosis over the years [4], especially for carcinomas diagnosed in early stages, it seems even more important to avoid long-term side effects due to diagnostic procedures, i.e. tumor seeding.

While past data and did not show a relevant effect of needle tract seeding and following metastasis by percutaneous biopsy [12] [13], there is currently increasing evidence for a significant effect, especially in stage-I-tumors [15] [14]. It might be assumed that this could be driven by therapeutic advances leading to a longer overall survival and revealing overlooked consequences.

The median time to the appearance of tumor seeding was previously determined by 7 months [13], however case reports listed intervals up to 26 months after obtaining the tissue samples [22], which exceed the median lifespan after diagnosis estimated one year in general by german health authorities [20].

**Fig. 3:**
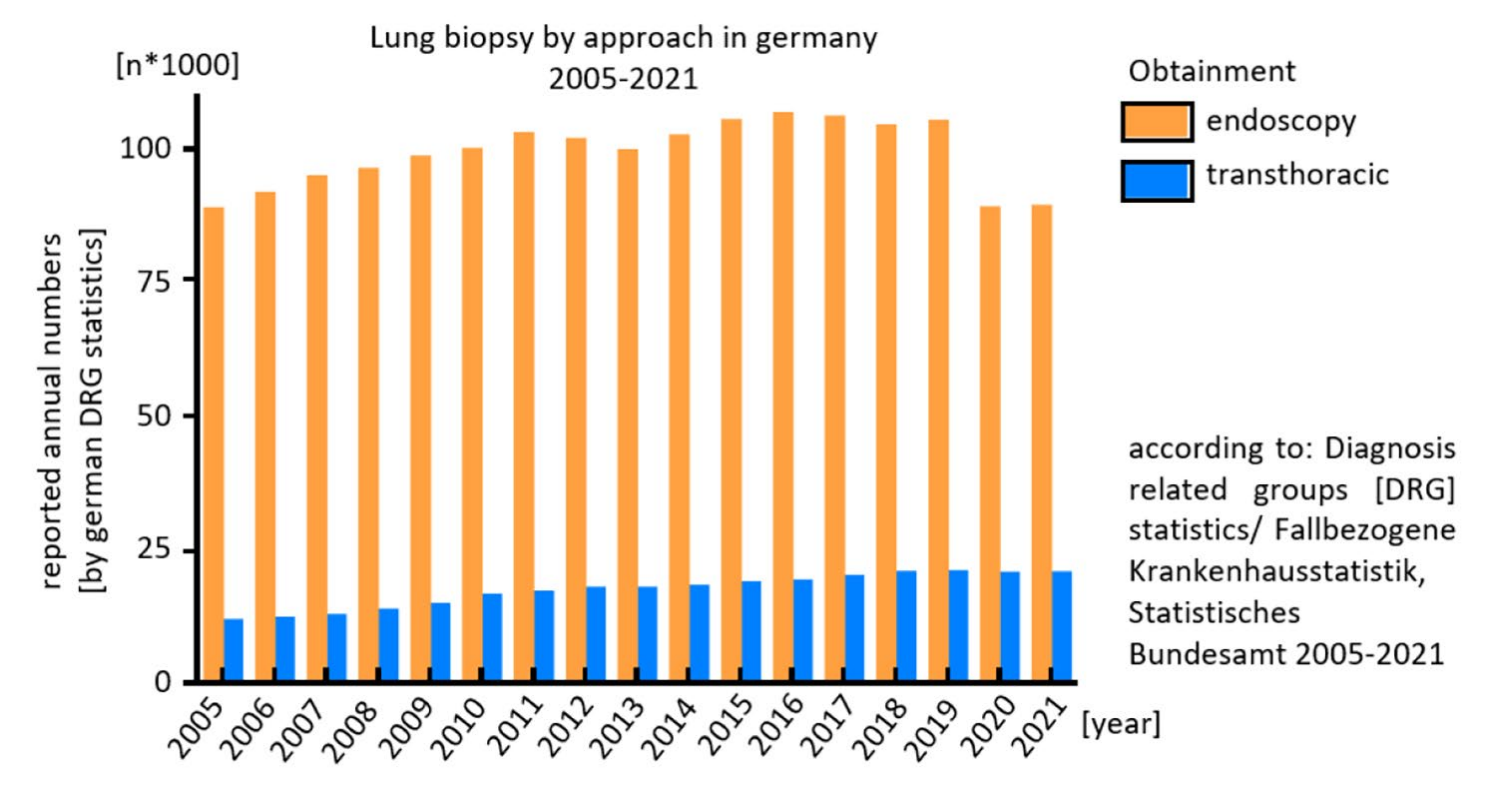
According to german diagnosis-coding-statistics, the number of annual transthoracic lung biopsies, mostly conducted CT-guided, almost doubled from 11.197 in 2005 to 20.213 in 2021. In opposite, bronchoscopic tissue obtainment stagnated lastly with a relevant cut during beginning Covid-19 pandemic. However in absolute numbers it still remains as the most applied procedure. (Statistisches Bundesamt (Destatis), Gesundheit: Fallpauschalenbezogene Krankenhausstatistik (DRG-Statistik) Operationen und Prozeduren der vollstationären Patientinnen und Patienten in Krankenhäusern (4-Steller), 2005-2021, Wiesbaden, published online, [cited November 11^th^ 2022])

Current data [14] [15] and our case report indicate that late diagnostic complications may become more frequent in the future. In Germany, CT-guided lung biopsy has almost doubled from 2005 to 2021, while bronchoscopic tumor sampling declined probably by the Covid-19 pandemic (Fig. 3). Due to the increasing implementation of lung cancer screening programs worldwide, each patient with pulmonary nodules should be discussed in a multidisciplinary tumor team [23]. Next to currently assessed criteria like intrapulmonary localization and size, also the long-term risks of bioptic procedures should be taken into account. Video-assisted thoracoscopy and probatory resection might be considered as a third option [10] but result in higher patients’ burden and resource consumption.

Probably, it might be possible to determine a risk constellation by tumor phenotype in imaging. For example, a meta-analysis by Li et al. reported that sub-pleural lesions of primary tumors could be an indicator for pleural local recurrence, even if there was no general association between CT-guided lung biopsy and overall tumor recurrence [24] [25]. This could correlate to a biological phenotype meliorating soft tissue invasion, just as seen in our case by developing an independent second metastasis below in spine.

However, there are also limitations regarding the presented case and related findings in the literature. Firstly, even if tumor seeding must be seen as a notable adverse event, the impact on patient-relevant outcomes like quality of life and overall survival is still poorly investigated. Only one study concucted by Moon et al. showed the absence of a relation between CT-guided biopsy and overall recurrence-free survival [26].

Our patient has been initially diagnosed with brain metastasis, and even if there is no information on final cause of death, the soft tissue metastasis next to the biopsy channel caused pain, but a substantial change in prognosis is unlikely.

Retrospective analyses, however, cannot cover the real risk situation for tumor seeding after CT-guided biopsy to a sufficient extent. Despite the knowledge of the need for a long follow-up period, patients with CT-guided puncture should be followed up in controlled study settings to gain deeper understanding for late complications.

This seems of high clinical relevance due to the ongoing advances in oncologic therapy leading to better survival outcomes independent of tumor stage [27].

## Data Availability

Not applicable.

## Declarations

### Reporting guidelines

This case report was written regarding CARE guidelines.
